# Twitter as a Sentinel in Emergency Situations: Lessons from the Boston Marathon Explosions

**DOI:** 10.1371/currents.dis.ad70cd1c8bc585e9470046cde334ee4b

**Published:** 2013-07-02

**Authors:** Christopher A. Cassa, Rumi Chunara, Kenneth Mandl, John S Brownstein

**Affiliations:** Harvard Medical SchoolBrigham and Women's Hospital; Assistant Professor of Pediatrics, Harvard Medical School and Harvard-MIT Division of Health Sciences and Technology, Harvard, USA

## Abstract

Immediately following the Boston Marathon attacks, individuals near the scene posted a deluge of data to social media sites. Previous work has shown that these data can be leveraged to provide rapid insight during natural disasters, disease outbreaks and ongoing conflicts that can assist in the public health and medical response. Here, we examine and discuss the social media messages posted immediately after and around the Boston Marathon bombings, and find that specific keywords appear frequently prior to official public safety and news media reports. Individuals immediately adjacent to the explosions posted messages within minutes via Twitter which identify the location and specifics of events, demonstrating a role for social media in the early recognition and characterization of emergency events. *Christopher Cassa and Rumi Chunara contributed equally to this work.

## Introduction

On April 15 at 2:49pm EDT, two bombs improvised from pressure cookers exploded on the sidewalk near the finish line of 117^th^ Boston Marathon. 264 patients were transported to 19 emergency departments throughout Boston^1^.

Public health authorities provided alerts to regional emergency departments approximately nine minutes after the explosions, just as ambulances left the scene^2^ via the Massachusetts Emergency Preparedness Bureau (MA EPB) Health and Homeland Alert Network (HHAN). The Massachusetts Central Medical Emergency Direction (CMED) Center also began communicating to individual hospitals by radio in the minutes prior. We sought to measure the timing of social media reports in relation to those issued through these official emergency response channels.

Social media and other mobile platforms enable individuals to post messages along with specific geographic information. These messages can help track infectious disease outbreaks,^3^ aid in natural disaster response^4^ and provide insight into conflicts^5^, where data collected through official reporting structures can take weeks to collect and analyze. Twitter streams are routinely used by high frequency trading applications to rapidly assess external factors that may affect market conditions^6^ and used in marketing to assess consumer response in real-time to advertisements.^7^ Data from informal media are typically available in near real-time and can be combined with geographically encoded news media reports and traditional data sources to improve surveillance.^8^ Here, we characterize the early social media response to a geographically-constrained, rapidly evolving critical situation.

## Geo-localized social media trend analysis

Our analysis was based on the set of Twitter postings with geolocation data (latitude and longitude) freely available via the Public Twitter API.^9^ To increase specificity, we narrowed the radius of the tweets to 35 miles from the Boston Marathon finish line. We observed messages containing the word stems: ‘explos*’ or ‘explod*’, just 3 minutes after the explosions (Figure 1). When adding words beginning with ‘bomb’, a picture quickly emerges of an incident warranting further exploration.

While an increase in messages indicating an emergency from a particular location may not make it possible to fully ascertain the circumstances of an incident without computational or human review, analysis of such data could help public safety officers better understand the location or specifics of explosions or other emergencies. Figure 1 illustrates the timeliness of the social media data, along with initial Twitter postings about the event from pertinent national and local news sources (CNN, the Associated Press, Boston WCVB) and with electronic messages from the MA EPB HHAN. Social media messages directly from individuals on the ground were timely, followed closely by validated public health alerts; messages from news sources followed both of these.

**Cumulative time series of tweets from within a 35 mile radius of the Boston Marathon finish line selected using the stems “explod*”, “explos*” and “bomb*” after the bombings at 2:49.**
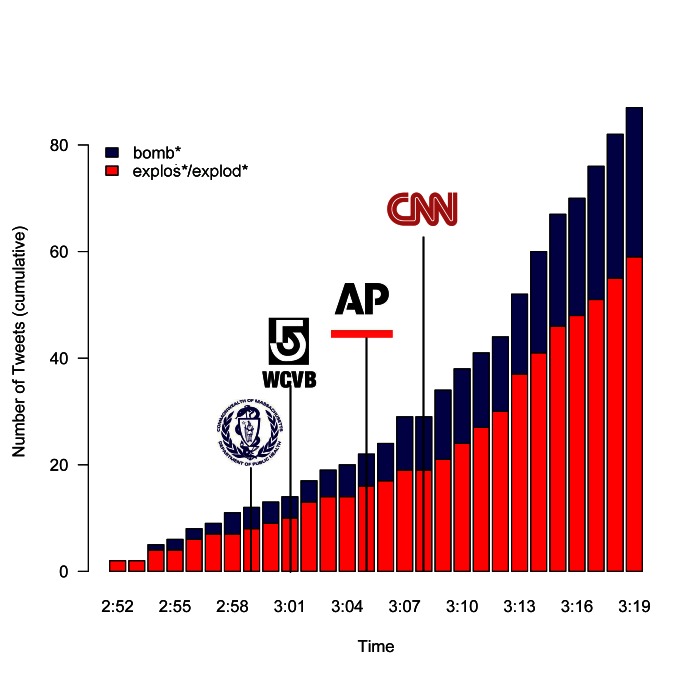
Public health officials alerted regional emergency departments via the HHAN at 2:59. Reports from news stations such as WCVB, the Associated Press, and CNN followed shortly after.

The baseline level of messages with these included keywords is very low in this area. For the stems ‘explos*’ and ‘explod*’, there was only one other message the day prior to the explosions in the same geographic radius.

## Geolocation and characterization of the event

Within the first 10 minutes of the bombings, many of the observed messages were from the immediate vicinity of the finish line (Figure 2).

**Public Twitter messages selected using the stems “explod*” and “explos*” in the immediate vicinity of the Boston Marathon finish line from the first 20 minutes after the bombings.**
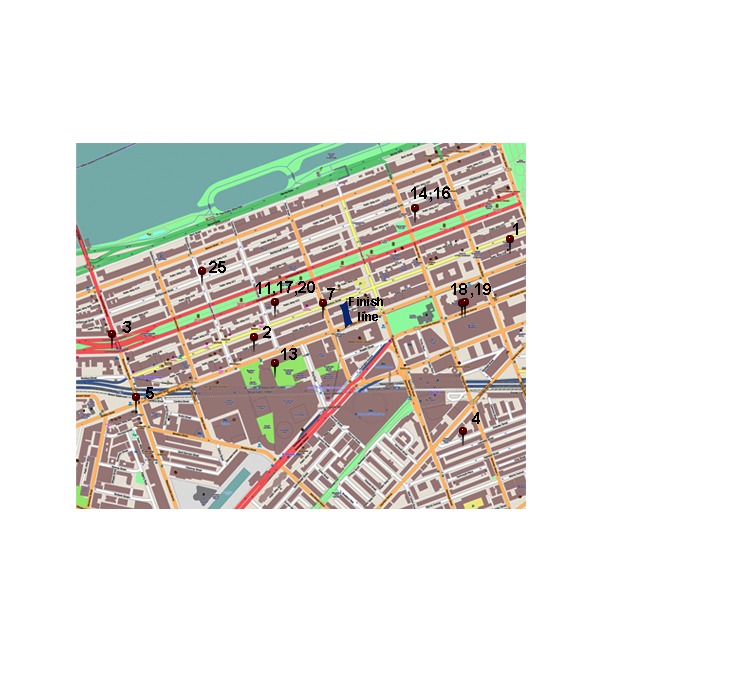
These messages were selected for inclusion because of their proximity to the event and content of these postings. Source OpenStreetMap.

Because of their proximity to the event and content of their postings, these individuals might be witnesses to the bombings or be of close enough proximity to provide helpful information. These finely detailed geographic data can be used to localize and characterize events assisting emergency response in decision-making. In the Supplementary Materials, we include the text of the Twitter messages from individuals (SM Table 1) and (SM Table 2), and Twitter postings from select news sources (SM Table 3). We have redacted expletives and personal identifiers.

## Discussion

Each year, the Boston Athletic Association provides a medical tent near the finish line of the Boston Marathon as well as a strong security and media presence. Hence, first responders, law enforcement and reporters were already present near the explosions, and were able to respond to the injuries, activate the emergency response system, and begin the investigation. In other situations, crowd-sourced information may uniquely provide extremely timely initial recognition of an event and specific clues as to what events may be unfolding--e.g. “area of 671 Boylston St.”, “hundreds hurt...bloody”—that could be used to tailor and refine the response.^2^ Here we described how data from Twitter provided localization and characterization of the Boston Marathon explosions.

Caution in the use of social media reports is warranted, however. While social media data can provide timely insight into events as they unfold, they may also produce false positive reports with negative effects, as illustrated by a powerful example from the financial sector. In the recent “flash crash,” a spurious Twitter report of a White House attack by the Associated Press was promulgated over 4,000 times.^6^ This led automated financial systems to take rapid – but inappropriate – action, which was quickly reversed.^10^

Classification strategies and filtering approaches that have been developed for disease surveillance,^8^^,^ 11 ^,^ 12 ^,^ 13 geospatial cluster identification,^14^ and crime tracking^15^ may help refine the sensitivity and specificity as well as classify postings into relevant categories such as personal, informative and other.^16^ Additionally, by comparing newly observed data against temporally adjusted keyword frequencies, it is possible to identify aberrant spikes in keyword use. The inclusion of geographical data allows these spikes to be geographically adjusted, as well. Prospective data collection could also harness larger and other streams of crowdsourced data, and use more comprehensive emergency-related keywords and language processing to increase the sensitivity of this data source. The analysis of multiple keywords could further improve these prior probabilities by reducing the impact of single false positive keywords derived from benign events.

In the wake of the explosions, Twitter became a news source for many individuals, which allowed unvetted information to enter the public sphere. For example, in our keyword analysis, we see two tweets that blamed individuals from Korea for the explosions (SM Table 1 and SM Table 2). This misinformation -- which may be posted in a practically anonymous fashion -- is difficult to correct or expunge once it has been cited by the media or shared extensively on social media platforms.

Given this risk, the sensitivity and specificity of notifications must be optimized using the perceived cost of intervention (e.g. unnecessary investigation) and the opportunity cost of not reacting when appropriate (e.g. delay in care). In events that unfold over a longer period of time, or require more in-depth investigation, it is possible that the dangerous impact of false positives may be reduced, or that the data may be weighted to mitigate unwanted consequences.

There is a real opportunity to make use of Twitter streams and other social media data to expedite public health, safety, or medical response in crises. Approaches to actively survey social media to complement traditional approaches to situation awareness after emergency events should be developed which integrate with existing analysis and alerting infrastructure.

## Appendix 1

### Supplementary Table 1

**Table:** Keywords beginning with stem word ‘explo’ within first 20 minutes

There was just an explosion at Copley...seriously. Wtf...
Two explosions just rocked the finish line of the Boston Marathon. Sirens galore. People running in fear. Wonder what happened.
Did something just explode… twice in town? @*****
Just heard two large explosions at the #bostonmarathon
2 explosions just happened here on boylston st. #boston #bostonmarathon
first something exploded and then they made everyone clear the way so an ambulance could go through... #whathappened?
can someone tell me what that explosion was!? #boston #bostonmarathon
Holy **** there was explosions in Copley.
@***** @***** RT: @***** Large explosion on the Boston Marathon route, area of 671 Boylston St. Possibly 60 people injured
That explosion in Boston just shook my house
Multiple explosions @ Boston Marathon
BREAKING NEWS: Two powerful explosions detonated in quick succession right next to the Boston Marathon finsh line this afternoon.
There was just a ****ing explosion on boylston street
Also what exploded
And then this. Why? “@*****: RT @*****: Live video from scene of Boston Marathon explosion http://t.co/mYxdURqnT8
what exploded
BREAKING NEWS: Two powerful explosions detonated in quick succession right next to the Boston Marathon finsh line this afternoon.
I am fine, I was literally by where the explosions happened less than 1 hour ago, my office is right by finish line
@***** I heard a loud BOOM and they are saying there were two explosions, seems many hurt
#BostonMarathon explosion, why why why !!!! http://t.co/eAGDM8F8wH
That explosion was definitely set up ....
were the explosions terrorist attacks?
"@*****: This explosion is scary
Jesus. MT @*****: Aerial shot of the finish line were the explosions happened. (Fox #Boston) http://t.co/8i86l45XlO
Multiple people are injured near the Boston Marathon finish line after two explosions. The #BostonMarathon has been stopped.
What explosion lol
I hope everyone is okay at the marathon from the explosion
Holy ****. Prayers go out to those injured/effected by the explosions at the finish line. Hopefully nothing else happens.
2 explosions at Boston marathon
So scared. Stuck in Boston because my car won't start and probably won't be able to get out bc of the explosions. Hope everyone is okay.
Bomb just exploded at the Boston Marathon #bostonmarathon
Two explosions @ the finish line of the Boston Marathon....horrific scene, hundreds hurt...bloody. On no! This is terrible.
My aunt literally just said she think North Korea is responsible for the Boston Marathon explosion
Two explosions by the finish line of #bostonmarathon
#BREAKING 2 explosions at Boston marathon finish line.
Some of these Boston Marathon explosion pics are gruesome.
**** my moms on the train I'm gunna explode
And then the city started to explode and we couldn't get out...........
@***** two explosions. Some major injuries.
Watching this explosion on the news is reminding me of 9/11
Just heard two huge explosions in Boston. What is going on?

## Appendix 2

### Supplementary Table 2

**Table:** Keywords beginning with stem word ‘bomb’ within first 20 minutes

Two bombs just went off on boylston.....
Holy **** did a bomb just go off????
Sounds like a few bombs went off across the street
Two bomb sounds and a building on fire on one of the biggest days in Boston... Coincidence I think ****ing NOT
WTF the boston marathon just got bombed
Marathon got bombed wtf, thats right near my work
Koreans bombed Boston spread the word
I can't believe I just witnessed a bombing #blessedtobesafe
Two men had bombs strapped to themselves and they both went off. Everyone is scrambling.
I hate that my mom works at the airport I always think people are gunna bomb it
@***** it was 2 bombs! You okay?
Omg...omg....I was standing right down from where the bomb went off earlier
A bomb went off at the marathon?
O **** they calling bomb squad and FBI **** got real
Hoards of people being directed away from Copley Square where a #bomb went off at the #BostonMarathon… http://t.co/Aq6v2AqAqs
Two bombs just went off in Boston!? #wtf #BostonMarathon
@***** bombs went off in boston I have so many friends here who are in the area I'm so worried.
Two bombs went off during the marathon? #****edup
@***** I am yes, we only saw two big puffs of smoke and they sounded a lot like bombs

## Appendix 3

### Supplementary Table 3

**Table:** News and public safety source initial messages regarding the bombings

**3:01 @WCVB Explosion reported near Boston Marathon finish line**
**3:05 @AP BREAKING: Two explosions at the finish line of the Boston Marathon result in injuries -BW**
**3:08 @CNN There has been at least one explosion near the Boston Marathon finish line, according to CNN affiliate WCVB. Details on @CNN TV now.**
**3:39 @boston_police Boston Police confirming explosion at marathon finish line with injuries. #tweetfromthebeat via @CherylFiandaca**
**3:47 @bostonmarathon There were two bombs that exploded near the finish line in today's Boston Marathon. We are working with law…http://fb.me/1HdL4nLXX**
